# A Double-Edged Sword: The Role of Prior Knowledge in Memory Aging

**DOI:** 10.3389/fnagi.2022.874767

**Published:** 2022-05-10

**Authors:** Xi Chen, Leah Varghese, William J. Jagust

**Affiliations:** ^1^Helen Wills Neuroscience Institute, University of California, Berkeley, Berkeley, CA, United States; ^2^Molecular Biophysics and Integrated Bioimaging, Lawrence Berkeley National Laboratory, Berkeley, CA, United States

**Keywords:** associative memory, congruency, knowledge, memory aging, semantic memory, schema, object–scene perception

## Abstract

**Introduction:**

People accumulate knowledge throughout their lifespan and the accumulated knowledge influences how we encode and retrieve information in memory processing. This study aims to investigate the role of knowledge in associative memory across the adult lifespan, and specifically examines the effects of two material properties that interact with prior knowledge: congruency – whether the material is congruent with people’s prior knowledge, and ambiguity – whether the material is ambiguous to interpret based on prior knowledge.

**Method:**

273 participants (aged 22–70 years old) completed an incidental memory task online. Participants were shown pictures depicting an object in a scene and judged if the object was likely or unlikely to be in the particular scene. Later, in the recognition test, participants were asked to identify if the exact picture was presented earlier. The pictures were manipulated to have varying levels of congruency, meaning that some depicted likely object–scene pairs and some unlikely. We also measured how different the likely/unlikely judgment for each object–scene pair was across all participants to determine the ambiguity level of the object–scene pair: some were more likely to receive diverse responses across people, whereas others are unambiguously consistent (or inconsistent) with common knowledge shared by most people. We used mixed-effects logistic regressions to predict memory outcome for each trial as a function of age, age^2^, congruency/ambiguity, and their interactions.

**Results:**

The object–scene pairs perceived as congruent had higher hit rates than incongruent ones, as well as higher false alarm rates, especially in middle-aged and older people. Higher ambiguity was also related to both greater true and false memory, independent of age. Finally, the effect of ambiguity only emerged when the object–scene pair was perceived incongruent.

**Discussion:**

The results suggest that people rely on prior knowledge to process new information and that this reliance improves hit responses, but also induces false memories particularly for middle-aged and older people, suggesting a double-edged role of knowledge in associative memory and its disproportionate influence on memory aging. Over-reliance on knowledge in older adults, which has been suspected in other cognitive processes, may be one of the mechanisms underlying associative memory decrease in aging.

## Introduction

The study of memory aging has taken on particular significance over the past decades, because memory is a vulnerable domain in cognitive aging (Budson and Price, 2005; Brickman and Stern, 2009; Tromp et al., 2015), making it a promising candidate when trying to detect and predict significant cognitive changes in older people. In particular, associative memory, the ability to remember associations between individual items, is a crucial process of episodic memory and declines faster than memory for items (Dunlosky and Hertzog, 1998; Naveh-Benjamin, 2000; Naveh-Benjamin et al., 2003; Old and Naveh-Benjamin, 2008; Naveh-Benjamin and Mayr, 2018). The ability to remember associations does not stem from the mere recognition of individual items but rather demands memory of all components and, particularly, the relationship between them. Worse associative memory has been found to be related to differences in hippocampal structure and function (Shing et al., 2011; Carr et al., 2017), decreased network integrity (Ren et al., 2015), reduced neural specificity (Saverino et al., 2016), and early AD pathology (Rentz et al., 2011) in older people.

One important aspect of associative memory quality is how new information interacts with prior knowledge and how existing knowledge structure guides and facilitates the memory process (Hess, 1994; Greve et al., 2019). People accumulate knowledge throughout their lifespan, and this acquired knowledge about the world, also referred to as semantic memory (Yee et al., 2014; Renoult et al., 2019), influences how new information is perceived and integrated (Van Kesteren et al., 2012; Ghosh and Gilboa, 2014). For example, a picture depicting a scenario consistent with one’s schematic knowledge (e.g., a rubber duck in a bathroom) is often better encoded and more likely to be remembered later, than an incongruent scenario (e.g., a telephone in a bathroom; Bransford and Johnson, 1972; Van Kesteren et al., 2012; Brod and Shing, 2019). This superior memory performance for knowledge-congruent information is known as the memory congruency effect (Craik and Tulving, 1975; Moscovitch and Craik, 1976). It is believed to reflect a more elaborative encoding and facilitated retrieval using existing knowledge (Staresina et al., 2009; Ghosh and Gilboa, 2014), and is achieved *via* knowledge-dependent interactions between medial temporal lobe (MTL) and prefrontal cortex (PFC; Van Kesteren et al., 2012; Preston and Eichenbaum, 2013). Particularly, PFC is suggested to control the activation and selection of knowledge stored in semantic memory (Binder et al., 2009; Binder and Desai, 2011): it detects the congruency of the to-be-encoded information and resonates with existing knowledge represented in the neocortex (Spalding et al., 2015); the MTL, particularly the hippocampus, is responsible for incorporating and binding elements and making associations and is particularly active when the information is incongruent with existing knowledge (Diana et al., 2007; Renoult et al., 2019). Successful associative memory depends on the proper interactions between PFC and MTL memory systems.

Given the role of MTL and PFC in associative memory and how they change with age (Raz et al., 2005; Davis et al., 2008; Stawarczyk et al., 2020), vulnerabilities in associative memory may be related to how knowledge affects memory and reflects certain mechanisms underlying episodic memory aging. Indeed, some recent studies reported age-related differences in the role of semantic knowledge on associative memory (Hess and Slaughter, 1990; Gutchess and Park, 2009; Brod et al., 2013; Badham et al., 2016; Brod and Shing, 2019; Wynn et al., 2020; Aghayan Golkashani et al., 2021) and the majority have found that older adults appear to show a preserved or even stronger memory congruency effect than young adults, likely due to their higher reliance on prior knowledge to process knowledge-congruent information. For example, Wynn et al. (2020) showed participants pictures of objects in a scene and asked participants to find a certain object in the picture; sometimes the object was in an expected location (congruent), and sometimes the location was unexpected (incongruent). Participants were later presented the scene without any objects and asked to remember (1) where an object was in this scene, and (2) which object appeared in this scene. The authors found that people took less time to find target objects in the congruent condition and that this benefit was larger in older adults, suggesting that prior knowledge about the scene aided the visual processing, particularly for older adults. They also found that such reliance on knowledge enhanced people’s memory for the object location in the congruent condition: people remembered the location of the object better when the object was in an expected location. This, along with other findings, appears to suggest that knowledge-congruent information is more memorable, especially for older people.

However, some studies also found that participants, particularly older participants, had more false memories for congruent pairs (Roediger et al., 2001; Castel et al., 2007; Kleider et al., 2008; Umanath and Marsh, 2014). For example, a recent study (Brod and Shing, 2019) also used object–scene pairs, consisting of one object picture and one scene picture side-by-side, and examined the associative memory for knowledge-congruent (if the object and the scene “fit together”) and knowledge-incongruent (if the object and the scene “do not fit together”) pairs in children, young, and older adults. They found that the older group had a stronger memory congruency effect than younger adults, but also a higher tendency to commit knowledge-congruent errors, where older adults were most likely to misremember a new object–scene pair as “old” when the object and scene fit together, suggesting a negative influence of relying on knowledge in memory judgments. Taken together, these findings suggest that the role of prior knowledge in memory formation appears to be inconclusive but likely significant, especially in older adults who have lost cognitive resources for information processing but show relatively preserved knowledge (Park et al., 2002).

These previous findings provide an important foundation for recognizing the important and complex influence of knowledge in memory and aging. However, prior research exhibits two major shortcomings regarding the generalization of the conclusions: (1) the definition of congruency/incongruency is only based on the experimenter’s judgment, and (2) the extreme age groups under study. Regarding the former, almost all previous studies manipulated congruent and incongruent associations based on the experimenter’s definition. However, knowledge accumulates and changes throughout the lifespan (Ackerman, 1996; Park et al., 2002), and varies across individuals (Ackerman et al., 2001). The present study asks participants to judge whether the presented pairing of objects and scenes is congruent or incongruent with their own knowledge and examines their subsequent memory as a function of their own judgments. Additionally, we included a *post hoc* measure of “ambiguity” to reflect how variable the responses of the judgment are across all participants for each object–scene pair: a pair with a high ambiguity index represents scenarios where people have more diverse interpretations, and a pair with a low ambiguity index is perceived similarly and unambiguously across people. This is an important property of the material that has rarely been studied but should be carefully considered. For the latter, studies on knowledge and memory aging have been mostly focusing on studying the extreme groups contrasting young and older participants, even though cognitive aging, as well as its underlying mechanisms, is well recognized as a continuous, and likely non-linear, process (Verhaeghen and Salthouse, 1997; Fjell et al., 2010).

In summary, the present study investigates one manipulation variable (congruency) and one *post hoc* measure (ambiguity) to examine the role of knowledge in remembering object–scene pairs across the adult lifespan. We focus on these two properties because they are inherent in experimental materials of many memory studies and are central to associative memory. We examine both the positive and negative influence of congruency and ambiguity of test stimuli on memorability. The influence of material congruency and ambiguity on memory in people of different ages is not only important for understanding mechanistic differences in the role of knowledge for memory between young and older adults but also necessary to assess potential material bias in certain memory assessments in older people. We use a lifespan sample including young, middle-aged, and older adults to examine the dynamic change of the effect of knowledge on memory and whether some memory deficits in older adults may be explained by the use of knowledge in memory. This also allowed us to measure any non-linear, age-related effect that is not possible to detect without a middle-aged sample. Finally, this experiment uses a newly designed paradigm that integrates object and scene items, rather than showing them to participants separated, side by side, or superimposed, which is more common in associative memory studies (Dulas and Duarte, 2016; Henson et al., 2016; Robin and Olsen, 2019; van Kesteren et al., 2020). This creates more ecologically-valid materials holistically depicting an object in a scene. In addition, we used three different manipulations of object–scene pair lures (old object new scene, old scene new object, old object and old scene but new location) that challenge the precision of specific memory subprocesses, which has rarely been examined in previous studies.

## Materials and Methods

### Participants

Participants were recruited from the online crowdsourcing platform Amazon Mechanical Turk (Mturk)^1^ using CloudResearch (Litman et al., 2017). The experiment was hosted *via* Pavlovia.^2^ A total of 283 participants completed the task, among which 273 were approved as valid data – 10 were rejected due to low data quality (see *Data Quality Assurance* section for details). The 273 participants (age range 22–70 years old; see Supplementary Figure 1 for age distribution) included 157 females (57.7%), 113 males (41.5%), and 3 missing sex information; 210 White (77.2%), 29 Black/African American (10.7%), 3 American Indian/Alaska Native (1.1%), 20 Asian/Pacific Islander (7.4%), 8 other (3%), and 2 missing race information. The average education was 15.26 years. All participants gave informed consent for their participation by pressing “y” on the consent form. Participants were compensated $10 for their participation. The study was approved by the Institutional Review Board at the University of California, Berkeley.

### Materials and Procedure

**FIGURE 1:**
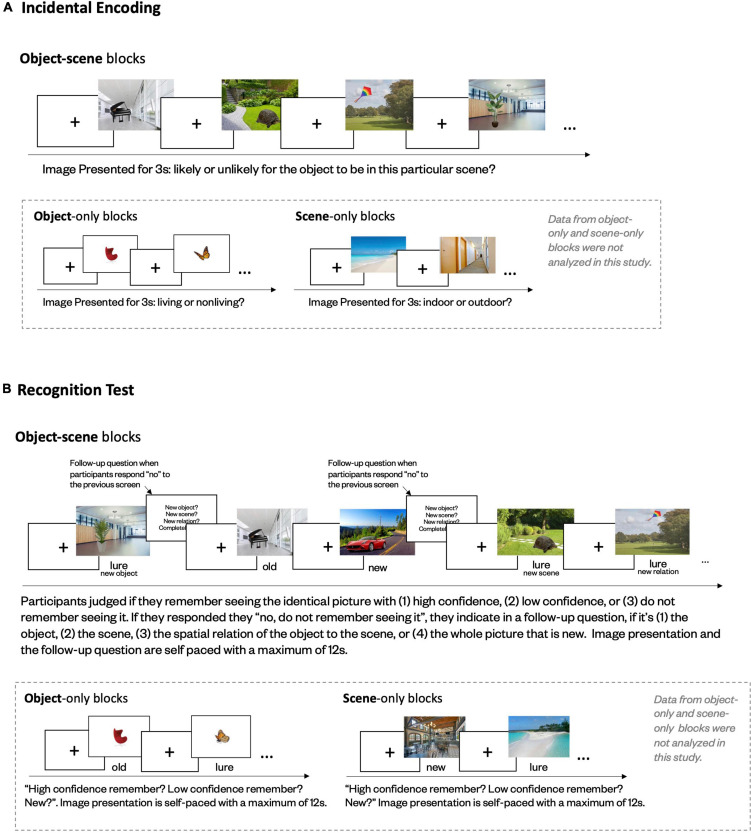
Experimental paradigm for encoding **(A)** and retrieval **(B)** phases in memory task.

**FIGURE 2:**
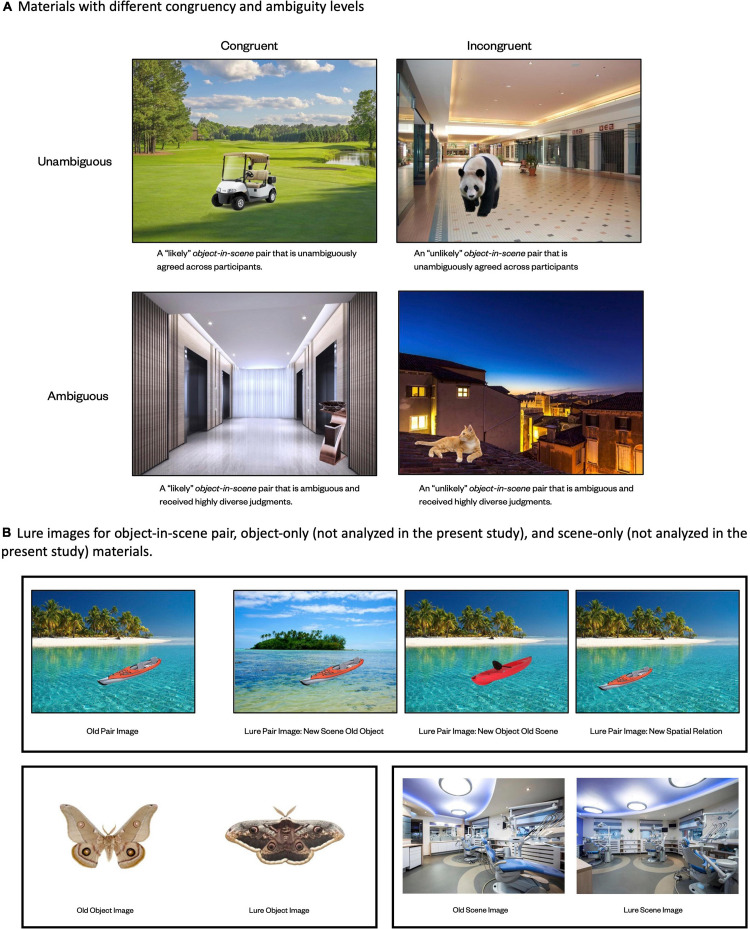
Examples of experimental materials used in the study. **(A)** Materials with different congruency and ambiguity levels. **(B)** Lure images for object–scene pair, object-only, and scene-only materials. Data of object-only and scene-only blocks were not analyzed in this study.

We used a newly developed memory task to assess memory with incidental encoding (Figure 1A). Materials being studied included images of *objects*, *scenes*, and *object–scene pairs* (Figure 2). During encoding, participants were shown 96 *object*, 96 *scene*, and 192 *object–scene pair* images, presented in 32 blocks – eight *object* blocks, eight *scene* blocks, and 16 *object–scene pair* blocks – with 12 trials in each block. During each trial, participants were first shown a fixation cross jittered for 1–3s, followed by 3s of the image. Participants were instructed to indicate, by pressing the “F” or “J” keys, if the object depicted a living or non-living object in the object blocks, if the scene depicted an indoor or outdoor scene in the scene blocks, and if the object appeared to be likely or unlikely to be in the particular scene in the object-in-scene pair blocks. At the start of each block, instructions and key mappings for the judgment questions were shown. The order of the blocks and trials was randomized. The present study only analyzed data from the *object–scene pair* blocks, where congruency judgments were made.

Among all the materials, half of the objects were living and half were non-living; half of the scenes were indoor and half were outdoor; roughly half of the object–scene pairs were designed to be likely and half were unlikely (Figure 2A). The inter-rater reliability of the likely/unlikely manipulation between two experimenters was high, with Cohen’s Kappa of 0.74 and 87% agreement. Since the likely/unlikely judgment for object–scene pairs was intended to be subjective, responses can vary across participants based on their prior knowledge and interpretation, but no pairs were bizarre or impossible. We checked if likely/unlikely judgment could vary systematically with age and found no relationship between one’s age and their judgment (*r* = 0.04, *p* = 0.479). We additionally generated a *post hoc* index, termed *ambiguity*, to reflect how variable the judgment was for each object–scene pair across participants. It is calculated based on the proportion of participants with differential answers, ranging from 0 to 0.5. Specifically, ambiguity = 1 – the majority proportion. For example, a pair consistently and unambiguously perceived as “unlikely” (or “likely”) across all participants receives a low ambiguity value of 0; a pair judged “unlikely” in half participants (and “likely” in the other half) has a high ambiguity value of 0.5; a pair judged “likely” in 80% participants receives a relatively low ambiguity index of 0.2 (Figure 2A). This measure was created to capture the variability in the interpretation of certain pairs and helps to delineate the influence of schematic ambiguity on memory, independent of whether the pair appears likely or unlikely. As a check, we compared the level of ambiguity between pairs judged incongruent and congruent and found no significant difference, *t*(190) = 1.51, *p* = 0.13.

After encoding, participants completed a 15-min survey on their demographic information. Following that, a surprise recognition test was given.

The recognition phase (Figure 1B) consisted of 24 blocks: six *object* blocks, six *scene* blocks, and 12 *object–scene pair* blocks, with 18 trials in each *object* and each *scene* block and 20 *trials* in each *object–scene pair* block. All images presented in the recognition phase were represented as either an identical old image from the encoding phase or a similar lure. Lures all had the same thematic and similar perceptual information, but visible differences from the old images (Figure 2B). For *object–scene pairs*, the lures had three forms: same object but in a lure scene, same scene but with a lure object, and same object and scene but in a different spatial relationship. New images, including 24 *object*, 24 *scene*, and 24 *object–scene pair* images, that were distinct from all old and lure images were also included. Whether a studied image was represented as old or lure (and what type of lure, for pairs) was randomly determined for each participant, with an equal chance of being old or lure (or different types of lures, for pairs).

During each recognition trial, participants were shown an image to judge if they remembered seeing the exact picture from the first part of the experiment with high confidence, with low confidence, or if they did not remember seeing the exact picture, by pressing “F,” “H,” or “J,” respectively. In addition, for *object–scene* pairs, if they indicated they did not remember seeing the exact picture, they were asked a follow-up question to indicate if they remembered the scene but not the object (“F”), the object but not the scene (“G”), both the object and the scene but in a different spatial relation (“H”), or if they didn’t remember seeing either the scene or object (“J”). The order of the blocks and trials was randomized. The instructions and key mappings for the memory questions were shown at the beginning of each block. In addition, the key mappings for each question stayed on the bottom of the screen during the presentation of the image.

### Data Quality Assurance

We employed several procedures to ensure data quality and a total of 10 participants were rejected after careful data quality checking due to low data quality. First, the task was only available through CloudResearch to a pool of “high-quality” participants who had no history of dishonest participation with fraud detection (Litman et al., 2017). CloudResearch also checks to identify suspicious VPN usage and blocks duplicate IP addresses to prevent the same participant from completing the survey again. Second, before the start of the task, 12 practice trials were administered. Participants with lower than 75% accuracy for the study judgment were required to read the instructions and complete the practice again until they passed it. This was done to make sure that participants understood the instructions. Third, participants were informed that their accuracy would be recorded and assessed in the study and that they were expected to always pay full attention. And throughout the experiment, multiple nonsensical check questions were included. These procedures were done to ensure that participants paid full attention at all times. Data with more than two check failures were rejected. Finally, after the participant completed the task, we carefully inspected each participant’s data, and multiple factors were taken into consideration to decide if the data would be approved. We reviewed their study judgment response during encoding, time to complete the survey, number of trials with reaction time under 200ms, and their optional voluntary feedback after task completion. The 10 low-quality participants were removed because they had more than two check failures and/or they voluntarily indicated their data should not be used.

### Statistical Analysis

Since we were interested in the role of knowledge in memory, the present study focused on analyzing the data from *object–scene pair* blocks where congruency and ambiguity were measured. We focused on the performance of the first recognition judgment in *object–scene pair* blocks, in which participants indicate if they remember seeing the *object–scene pair* image. We analyzed participants’ responses trial-by-trial using mixed-effects logistic regressions. To examine the effect of congruency, we used age, age^2^, congruency judgment response (yes/no), and their interactions to predict whether the memory outcome was a high-confidence hit (i.e., high-confidence yes response for an old trial), with intercepts of subject and image as the random effects. We *a priori* included age^2^ to capture any non-linear age-related change in memory. Using the same predictors, we also examined their prediction on high-confidence false alarm (i.e., high-confidence yes response for a lure trial). This allowed us to determine the effects of congruency on true and false memory separately. To explore any potential effect of congruency on memory discrimination, we used signal detection theory analysis and separately calculated the memory discriminability (*d’*) for the pairs judged as congruent and incongruent for each individual. We then used age, age^2^, congruency, and their interactions to predict *d’*, with the subject intercepts as the random effect.

Next, we examined the effect of ambiguity on memory. As detailed in the methods, an ambiguity index was calculated for each pair image based on the percentage of participants with different likely/unlikely judgments from the majority. We then centered the variable so that a higher, positive value indicated that the pair received more variable judgments among all participants, and a lower, negative value indicated a consensus with little ambiguity. We performed two mixed-effects logistic regressions using age, age^2^, the ambiguity index, and their interactions to predict high-confidence hit and high-confidence false alarm, separately, with the subject intercept as the random effect.

Finally, we examined the independent and interactive effects of congruency and ambiguity in the same model. We first used age, age^2^, congruency judgment, ambiguity index, and their interactions to predict high-confidence hit and high-confidence false alarm, separately, with the subject intercept as the random effect. Due to the complexity of the model, this initial model did not converge. We, therefore, removed age^2^ and its interaction terms and only included linear age effects because age^2^ was the non-significant polynomial predictor of the highest order, which is first considered to be removed when the model appears to be over-complex. This updated model successfully converged. We report results from this final model that included age, congruency judgment, ambiguity index, and their interactions as the predictors, with the subject intercept as the random effect.

All statistical analyses were conducted using R (v4.1.0) with *lme4* (Bates et al., 2014) and *interactions* (Long, 2019). All continuous variables were mean-centered to minimize multicollinearity. We also examined the variance inflation factor (VIF) for all variables and found all VIFs were smaller than 5, suggesting little evidence of collinearity (James et al., 2013).

## Results

**FIGURE 3:**
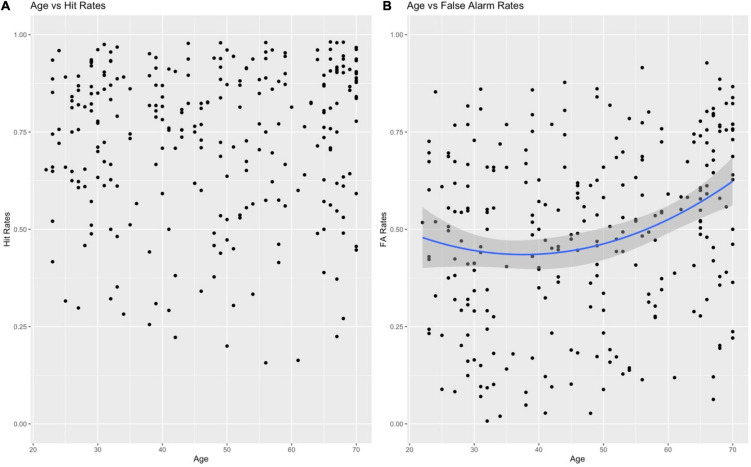
The relationship between age and memory performance – hits and false alarm rates. People at different ages performed similarly in hit rates **(A)** but older age was significantly related to an accelerated increase in false alarm rate. **(B)** Shaded area represents 95% confidence intervals for the regression trend line.

**FIGURE 4:**
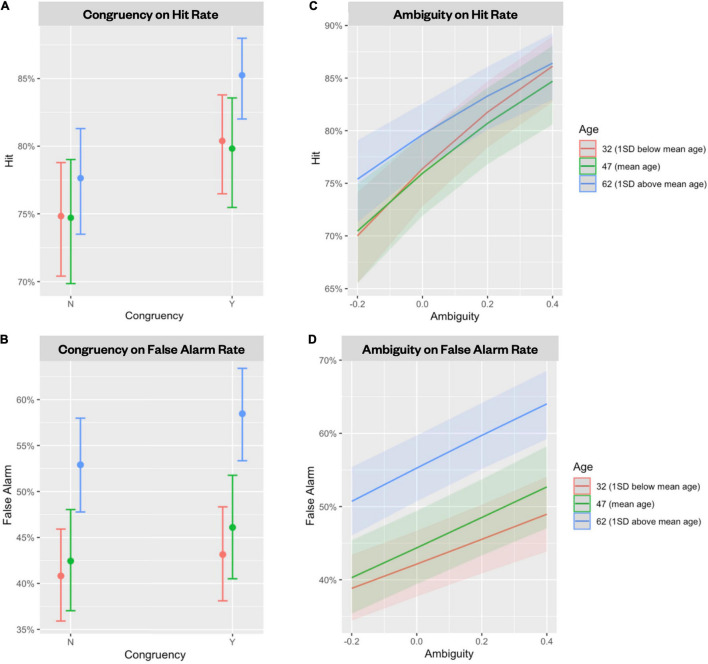
Effect of congruency and ambiguity on memory hit rate and false alarm rate. Congruent pairs were related to a higher hit rate (*p* < 0.001) particularly in people over 31 years **(A)**, as well as a higher false alarm rate (*p* < 0.001) in people over 45 years (*p* = 0.043). **(B)** More ambiguous pairs were related to a higher hit rate (*p* < 0.001) **(C)** and a higher false alarm rate (*p* < 0.001) **(D)** across all ages. Error bars and shaded areas represent 95% confidence intervals.

The first set of mixed effects logistic regressions examined the effect of age, age^2^, congruency judgment (yes/no), and their interactions in predicting high-confidence hit and high-confidence false alarms. We found that neither age nor age^2^ predicted high-confidence hits (*p*s > 0.30; Figure 3A), consistent with previous reports of no age difference in high confidence hits between young and older adults (e.g., Gutchess et al., 2005; Dennis et al., 2007). Congruency, on the other hand, significantly predicted high-confidence hits (β = 0.291, *p* < 0.001, Figure 4A); people were more likely to remember the object–scene pairs if they judged them to be congruent rather than incongruent. There was also a marginal age × congruency interaction (β = 0.061, *p* = 0.056) and a significant age^2^ × congruency interaction (β = 0.055, *p* = 0.033). The interactions occurred because the effect of congruency on memory was disproportionally stronger as age increased. Using the Johnson-Neyman procedure (Johnson and Fay, 1950; Aiken et al., 1991), we found the effect of congruency became significant after age 31 and continued to accelerate with increasing age. For high-confidence false alarms, the mixed-effects logistic regression revealed significant effects of age (β = 0.166, *p* < 0.001) and age^2^ (β = 0.079, *p* = 0.034), where older adults showed a significant and faster increase in false alarms as age increased (Figure 3B). We also found a significant effect of congruency judgment (β = 0.149, *p* < 0.001) in which people made more false recognitions for lures of congruent pairs than of incongruent pairs. There was also a significant interaction between age and congruency judgment (β = 0.043, *p* = 0.043; Figure 4B); people over 45 were more likely to have more false recognitions for congruent lures than for incongruent lures. Finally, we found age had a significant negative effect on *d’* (β = −0.073, *p* < 0.001) where older age was strongly related to decreased discriminability. Congruency had no effect on *d’* (*p* = 0.631) and the age-related interactions were not significant either (*p*s > 0.33). However, higher congruency had a marginal effect on *d*’ (*p* = 0.0546) once the non-significant age^2^ effects were removed (Supplementary Figure 2).

Next, to examine the effect of ambiguity on true and false memory, we performed two mixed effects logistic regressions using age, age^2^, ambiguity index, and their interactions to predict high-confidence hits and high-confidence false alarms, separately. Ambiguity index significantly predicted high-confidence hits (β = 1.405, *p* < 0.001); more ambiguous pairs were more likely to be correctly recognized (Figure 4C). No age-related interactions with ambiguity were found (*p*s > 0.20). For high-confidence false alarms, we found significant effects of ambiguity (β = 0.835, *p* < 0.001; Figure 4D), age (β = 0.179, *p* < 0.001), and age^2^ (β = 0.078, *p* = 0.028) where higher ambiguity and older age were both significantly related to higher false alarms. Neither age × ambiguity nor age^2^ × ambiguity interactions were significant (*p*s > 0.16).

**FIGURE 5:**
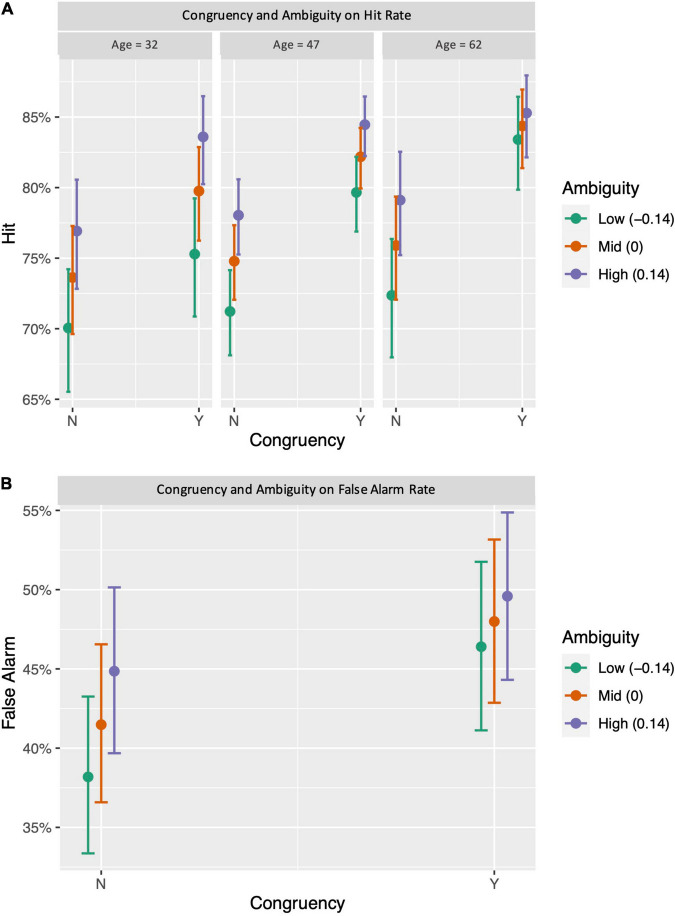
Interactive effects of congruency and ambiguity on memory hit rate and false alarm rate. **(A)** For hit rate, ambiguity effect diminished for pairs perceived as congruent in people above 58 years old. **(B)** For false alarm rate, ambiguity only appeared to affect memory for pairs perceived as incongruent. Error bars represent 95% confidence intervals.

Lastly, to examine the independent and interactive effects of congruency and ambiguity, we used mixed-effects logistic regressions with age, congruency judgment (yes/no), ambiguity index, and their interactions to predict high-confidence hits and high-confidence false alarms, separately. For high-confidence hits, we found that the effects of congruency (β = 0.441, *p* < 0.001), ambiguity (β = 1.292, *p* < 0.001) and the age × congruency interaction (β = 0.065, *p* = 0.040) all remained significant. The model also revealed a significant three-way interaction between age, congruency, and ambiguity (β = −0.459, *p* = 0.037). The interaction occurred because the influence of ambiguity diminished when the object–scene pairs were judged as congruent in older adults over 58 years (Figure 5A). Finally, the model examining the same effects on high-confidence false alarms showed significant effects of age (β = 0.162, *p* < 0.001), congruency judgment (β = 0.277, *p* < 0.001), and ambiguity (β = 1.007, *p* < 0.001), as well as significant age × congruency judgment (β = 0.035, *p* = 0.028) and congruency × ambiguity interactions (β = −0.652, *p* < 0.001). The congruency × ambiguity interaction occurred because ambiguity was only significantly related to greater false alarms for incongruent pairs (Figure 5B).

## Discussion

Knowledge is important for creating new memories, and we demonstrate in this study how prior knowledge enhances and also misleads memory in people, particularly of older age. We used two measures – congruency and ambiguity – to assess how each object–scene pair was perceived by people based on their prior knowledge. When relating those measures to subsequent memory performance in a sample across the adult lifespan, we found that congruency was related to an overall memory benefit, indexed by a marginally higher *d*’, and both higher true and false memory, especially greater false memory in middle-aged and older people. Ambiguity was also related to both higher true and false memory, but its effect was independent of age. Finally, it appears that the influence of congruency may “override” the ambiguity effect as ambiguity only leads to false memory when the pair was perceived incongruent (when knowledge is not available for interpretations) and ambiguity only predicted better true memory for incongruent pairs in older adults. Overall, the results suggest that processing information with the aid of one’s prior knowledge can be both beneficial and detrimental for memory. Older adults’ memory precision can still benefit from relying on prior knowledge, despite the stronger negative influence that increases with age. This finding may help to delineate the potential contributions of material properties to associative memory deficits commonly observed in aging.

The influence of congruency on memory has been inconclusive in the literature (Simonsmeier et al., 2022). Studies have revealed memory enhancement related to both congruent and incongruent conditions (Poppenk et al., 2010; Greve et al., 2019), and have found both positive and negative effects of congruency on memory (Umanath and Marsh, 2014; Brod and Shing, 2019; Webb and Dennis, 2020). Here, in this study across the adult lifespan, we present strong evidence for both higher recognition (after the age of 31 years) and more false memories (after the age of 45 years old) related to congruency at the same time. The use of a lifespan sample allowed us to demonstrate that knowledge improves hit responses beginning in young adulthood and further accelerates with increasing age, whereas the effect on false memory does not emerge until middle age. This highlights the complex influence of knowledge on memory (Van Kesteren et al., 2012; Ghosh and Gilboa, 2014) and suggests age may be a moderating factor in how knowledge affects different aspects of memory.

The stronger influence of and greater reliance on knowledge in older adults has been documented in other cognitive domains (Hedden et al., 2005; Mata et al., 2007; Chen et al., 2017). This has been interpreted as an adaptive process in aging: older people utilize the semantic knowledge increasing through their life when facing the decrease in their fluid cognitive resources (Park et al., 2002; Salthouse, 2003). Although this shift allows older adults to take advantage of the better-preserved crystallized semantic knowledge and thus to better recognize knowledge-congruent information with little performance deficit, it also creates an age-related susceptibility that results in more false memories. This may be due to older adults having a more generic encoding strategy (Koutstaal and Schacter, 1997), facilitated by their over-reliance on schematic knowledge, which makes them more susceptible to misleading information during retrieval. The age-related increase in the negative effect of knowledge may explain, to some extent, why older people are more prone to misinformation (Brashier and Schacter, 2020): accumulated knowledge and their increased reliance on knowledge offer a stronger basis for confirmation bias, which may lead to more knowledge-congruent errors.

The other novelty of the study is that we examined material ambiguity, or how differently across people a pair was perceived, and found that greater ambiguity was also related to both higher true positives and false memories. The effect of ambiguity on memory has not attracted much attention in memory and aging research. One may predict that greater ambiguity weakens the access to existing knowledge, leading to a reduced benefit from knowledge on memory (Van Kesteren et al., 2012). However, we found the opposite effect. Ambiguity may affect memory in other ways besides the interactions with existing knowledge, which in fact reflects the complex nature of memory process. We believe in our present study, ambiguous pairs likely represent ill-defined scenarios that require deeper levels of processing, leading to a better encoding quality and thus enhanced recognition (Craik and Tulving, 1975; Bock, 1978). Meanwhile, higher ambiguity also indicates that greater interpretation is needed, leading to a higher likelihood of intrusions (Tuckey and Brewer, 2003). The effect of ambiguity was stronger when the material was perceived to be incongruent with prior knowledge, suggesting a particular vulnerability of memory when the material does not easily align with one’s knowledge and the elaborative encoding may thus be dampened. The ambiguous, incongruent pairs may demand more individualized knowledge to decode, which is not the case, however, when the material is consistent with common knowledge at a higher level and processing can rely on a stronger and densely connected knowledge structure. This suggests that once people can successfully configure and register the pair with their existing knowledge structure, whether or not the material was ambiguous to decode and judge does no longer play a role in the memorability of the information. This interactive effect of ambiguity and congruency on true memory was particularly strong in older adults, suggesting that the congruency of the material appears to be a stronger determinant of older adults’ recognition.

Recent studies have suggested that the use of prior knowledge may affect the efficiency of the interaction between the PFC and MTL during encoding and retrieval (for reviews, see Gilboa and Marlatte, 2017 and Van Kesteren et al., 2012). Specifically, memory processing for knowledge-congruent items is believed to be largely mediated by PFC, whereas the MTL compares and configures the incongruent information with existing knowledge. Given MTL atrophy and dysfunction in older adults (Raz et al., 2005; Stawarczyk et al., 2020), it is likely that aging is accompanied by a disruption of the balance between the two memory systems, which leads to a bias toward knowledge-congruent processing. This bias may facilitate memory processing of knowledge-congruent information and compensate for age-related deficits (Umanath and Marsh, 2014), which could partly explain the lack of age-related differences in hit rates across many memory aging studies (Gutchess et al., 2005; Dennis et al., 2007). This bias, however, may also contribute to a tendency for gist-based, generalized processing in older adults (Koutstaal et al., 1999; Greene and Naveh-Benjamin, 2020), as well as a decreased inhibition of misleading information consistent with prior knowledge in older people (Lustig et al., 2007; Colombel et al., 2016). These processes together result in a lack of detailed, specific encoding that is necessary for rejecting highly similar lures during recognition. Future studies may investigate the interactions of the two memory systems as age increases to better understand the role of knowledge in memory aging.

Several limitations of the current study should be acknowledged. The cross-sectional nature of the study may limit our interpretation of the observed age-related differences. It is possible that older adults participating in this study used different strategies, which could also contribute to the over-reliance on knowledge we suggest in the study. Future longitudinal examinations may investigate whether this difference in the role of knowledge is a developmental change in aging or an individual characteristic that is present in highly selected older adults. Our study is also different from other traditional in-person studies where participants are recruited from local communities. Future studies may examine if the results can be replicated in typical, in-lab samples. Our current finding, nevertheless, suggests the generalization of the congruency effect on memory to this unique online setting. We also acknowledge that a comprehensive investigation including additional measures of knowledge and fluid abilities would be helpful to examine whether accumulated knowledge may be particularly supportive in older individuals facing declining cognitive resources. Another limitation of the study is that we only examined two particular material properties – congruency and ambiguity – that are known to interact with people’s prior knowledge (Koutstaal et al., 2003; Tulver et al., 2019; Wynn et al., 2020), and that ambiguity was *post hoc*, operationally defined based on all participants. Many other aspects of a stimulus may also affect the memorability of the information. Future studies may examine other material properties (e.g., knowledge domain), individual perceived material ambiguity, and the influence of lure type (new scene, new object, new relation) to study if and how we could minimize potential testing bias in cognitive aging research.

In conclusion, this study investigated how knowledge influences memory in people across the lifespan and found that knowledge-congruent pairs were more memorable, but also more likely to induce false memories, especially for middle-aged and older adults. Higher ambiguity was also related to both increased hits and more false memories, but only when the pair was perceived incongruent. We conclude that prior knowledge plays a double-edged role in memory and is increasingly influential with aging.

## Data Availability Statement

The raw data supporting the conclusions of this article will be made available by the authors, without undue reservation.

## Ethics Statement

The studies involving human participants were reviewed and approved by the University of California, Berkeley. The patients/participants provided their written informed consent to participate in this study.

## Author Contributions

XC designed the study, performed the statistical analysis, and wrote the first draft of the manuscript. LV collected the data, performed data checking and data cleaning, assisted with statistical analyses, and wrote sections of the manuscript. WJ contributed to the conception and design of the study and oversaw the study. All authors contributed to data interpretation, provided edits to the manuscript, and approved the submitted version of the manuscript.

## Conflict of Interest

The authors declare that the research was conducted in the absence of any commercial or financial relationships that could be construed as a potential conflict of interest.

## Publisher’s Note

All claims expressed in this article are solely those of the authors and do not necessarily represent those of their affiliated organizations, or those of the publisher, the editors and the reviewers. Any product that may be evaluated in this article, or claim that may be made by its manufacturer, is not guaranteed or endorsed by the publisher.

## Footnotes

1
https://www.mturk.com/

2
https://pavlovia.org/

## Supplementary Material

Supplementary Figure 1 Age distribution of the sample.

Supplementary Figure 2 Higher congruency marginally related to better memory discrimination.

## References

[B1] Ackerman P. L. (1996). A theory of adult intellectual development: process, personality, interests, and knowledge. *Intelligence* 22 227–257. 10.1016/s0160-2896(96)90016-1

[B2] Ackerman P. L. Bowen K. R. Beier M. E. Kanfer R. (2001). Determinants of individual differences and gender differences in knowledge. *J. Educ. Psychol.* 93:797. 10.1037/0022-0663.93.4.797

[B3] Aghayan Golkashani H. Leong R. L. F. Wong K. F. Chee M. W. L. (2021). Schema-driven memory benefits boost transitive inference in older adults. *Psychol. Aging* 36 463–474. 10.1037/pag0000586 33646803

[B4] Aiken L. S. West S. G. Reno R. R. (1991). *Multiple Regression: Testing and Interpreting Interactions*. Thousand Oaks, CA: SAGE Publications.

[B5] Badham S. P. Hay M. Foxon N. Kaur K. Maylor E. A. (2016). When does prior knowledge disproportionately benefit older adults’ memory? *Aging Neuropsychol. Cogn.* 23 338–365. 10.1080/13825585.2015.1099607 26473767 PMC4784494

[B6] Bates D. Mächler M. Bolker B. Walker S. (2014). Fitting linear mixed-effects models using lme4. *arXiv* Preprint arXiv1406.5823,

[B7] Binder J. R. Desai R. H. (2011). The neurobiology of semantic memory. *Trends Cogn. Sci.* 15 527–536. 22001867 10.1016/j.tics.2011.10.001 PMC3350748

[B8] Binder J. R. Desai R. H. Graves W. W. Conant L. L. (2009). Where is the semantic system? a critical review and meta-analysis of 120 functional neuroimaging studies. *Cereb. cortex* 19 2767–2796. 10.1093/cercor/bhp055 19329570 PMC2774390

[B9] Bock M. (1978). Levels of processing of normal and ambiguous sentences in different contexts. *Psychol. Res.* 40 37–51. 10.1007/bf00308462

[B10] Bransford J. D. Johnson M. K. (1972). Contextual prerequisites for understanding: some investigations of comprehension and recall. *J. Verbal Learning Verbal Behav.* 11 717–726. 10.1016/s0022-5371(72)80006-9

[B11] Brashier N. M. Schacter D. L. (2020). Aging in an era of fake news. *Curr. Dir. Psychol. Sci.* 29 316–323. 10.1177/0963721420915872 32968336 PMC7505057

[B12] Brickman A. M. Stern Y. (2009). *Aging and Memory in Humans.* New York, NY: Columbia University.

[B13] Brod G. Shing Y. L. (2019). A boon and a bane: comparing the effects of prior knowledge on memory across the lifespan. *Dev. Psychol.* 55:1326. 10.1037/dev0000712 30802088

[B14] Brod G. Werkle-Bergner M. Shing Y. L. (2013). The influence of prior knowledge on memory: a developmental cognitive neuroscience perspective. *Front. Behav. Neurosci.* 7:139. 10.3389/fnbeh.2013.00139 24115923 PMC3792618

[B15] Budson A. E. Price B. H. (2005). Memory dysfunction. *N. Engl. J. Med.* 352 692–699. 15716563 10.1056/NEJMra041071

[B16] Carr V. A. Bernstein J. D. Favila S. E. Rutt B. K. Kerchner G. A. Wagner A. D. (2017). Individual differences in associative memory among older adults explained by hippocampal subfield structure and function. *Proc. Natl. Acad. Sci.U.S.A.* 114 12075–12080. 10.1073/pnas.1713308114 29078387 PMC5692588

[B17] Castel A. D. McCabe D. P. Roediger H. L. III Heitman J. L. (2007). The dark side of expertise: domain-specific memory errors. *Psychol. Sci.* 18 3–5. 10.1111/j.1467-9280.2007.01838.x 17362368

[B18] Chen X. Hertzog C. Park D. C. (2017). Cognitive predictors of everyday problem solving across the lifespan. *Gerontology* 63 372–384. 10.1159/000459622 28273664 PMC5471120

[B19] Colombel F. Tessoulin M. Gilet A.-L. Corson Y. (2016). False memories and normal aging: Links between inhibitory capacities and monitoring processes. *Psychol. Aging* 31:239. 10.1037/pag0000086 27111523

[B20] Craik F. I. M. Tulving E. (1975). Depth of processing and the retention of words in episodic memory. *J. Exp. Psychol. Gen.* 104:268. 10.1037/0096-3445.104.3.268

[B21] Davis S. W. Dennis N. A. Daselaar S. M. Fleck M. S. Cabeza R. (2008). Que PASA? The posterior-anterior shift in aging. *Cereb Cortex* 18 1201–1209. 10.1093/cercor/bhm155 17925295 PMC2760260

[B22] Dennis N. A. Kim H. Cabeza R. (2007). Effects of aging on true and false memory formation: an fMRI study. *Neuropsychologia* 45 3157–3166. 10.1016/j.neuropsychologia.2007.07.003 17716696

[B23] Diana R. A. Yonelinas A. P. Ranganath C. (2007). Imaging recollection and familiarity in the medial temporal lobe: a three-component model. *Trends Cogn. Sci.* 11 379–386. 10.1016/j.tics.2007.08.001 17707683

[B24] Dulas M. R. Duarte A. (2016). Age-related changes in overcoming proactive interference in associative memory: the role of PFC-mediated executive control processes at retrieval. *Neuroimage* 132 116–128. 10.1016/j.neuroimage.2016.02.017 26879623

[B25] Dunlosky J. Hertzog C. (1998). Aging and deficits in associative memory: What is the role of strategy production? *Psychol. Aging* 13:597. 10.1037//0882-7974.13.4.597 9883460

[B26] Fjell A. M. Walhovd K. B. Westlye L. T. Østby Y. Tamnes C. K. Jernigan T. L. (2010). When does brain aging accelerate? dangers of quadratic fits in cross-sectional studies. *Neuroimage* 50 1376–1383. 10.1016/j.neuroimage.2010.01.061 20109562

[B27] Ghosh V. E. Gilboa A. (2014). What is a memory schema? a historical perspective on current neuroscience literature. *Neuropsychologia* 53 104–114. 10.1016/j.neuropsychologia.2013.11.010 24280650

[B28] Gilboa A. Marlatte H. (2017). Neurobiology of schemas and schema-mediated memory. *Trends Cogn. Sci.* 21 618–631. 10.1016/j.tics.2017.04.013 28551107

[B29] Greene N. R. Naveh-Benjamin M. (2020). A specificity principle of memory: evidence from aging and associative memory. *Psychol. Sci.* 31 316–331. 10.1177/0956797620901760 32074021

[B30] Greve A. Cooper E. Tibon R. Henson R. N. (2019). Knowledge is power: Prior knowledge aids memory for both congruent and incongruent events, but in different ways. *J. Exp. Psychol. Gen.* 148:325. 10.1037/xge0000498 30394766 PMC6390882

[B31] Gutchess A. H. Park D. C. (2009). Effects of ageing on associative memory for related and unrelated pictures. *Eur. J. Cogn. Psychol.* 21 235–254. 10.1080/09541440802257274 20161025 PMC2749510

[B32] Gutchess A. H. Welsh R. C. Hedden T. Bangert A. Minear M. Liu L. L. (2005). Aging and the neural correlates of successful picture encoding: frontal activations compensate for decreased medial-temporal activity. *J. Cogn. Neurosci.* 17 84–96. 10.1162/0898929052880048 15701241

[B33] Hedden T. Lautenschlager G. Park D. C. (2005). Contributions of processing ability and knowledge to verbal memory tasks across the adult life-span. *Q. J. Exp. Psychol. A* 58 169–190. 10.1080/02724980443000179 15881297

[B34] Henson R. N. Campbell K. L. Davis S. W. Taylor J. R. Emery T. Erzinclioglu S. (2016). Multiple determinants of lifespan memory differences. *Sci. Rep.* 6 1–14. 10.1038/srep32527 27600595 PMC5013267

[B35] Hess T. M. (1994). Social cognition in adulthood: aging-related changes in knowledge and processing mechanisms. *Dev. Rev.* 14 373–412. 10.1006/drev.1994.1015

[B36] Hess T. M. Slaughter S. J. (1990). Schematic knowledge influences on memory for scene information in young and older adults. *Dev. Psychol.* 26:855. 10.1037/0012-1649.26.5.855

[B37] James G. Witten D. Hastie T. Tibshirani R. (2013). *An Introduction to Statistical Learning.* Berlin: Springer.

[B38] Johnson P. O. Fay L. C. (1950). The Johnson-Neyman technique, its theory and application. *Psychometrika* 15, 349–367. 14797902 10.1007/BF02288864

[B39] Kleider H. M. Pezdek K. Goldinger S. D. Kirk A. (2008). Schema-driven source misattribution errors: remembering the expected from a witnessed event. *Appl. Cogn. Psychol.* 22 1–20. 10.1002/acp.1361

[B40] Koutstaal W. Reddy C. Jackson E. M. Prince S. Cendan D. L. Schacter D. L. (2003). False recognition of abstract versus common objects in older and younger adults: testing the semantic categorization account. *J. Exp. Psychol. Learn. Mem. Cogn.* 29:499. 10.1037/0278-7393.29.4.499 12924853

[B41] Koutstaal W. Schacter D. L. (1997). Gist-based false recognition of pictures in older and younger adults. *J. Mem. Lang.* 37 555–583.

[B42] Koutstaal W. Schacter D. L. Galluccio L. Stofer K. A. (1999). Reducing gist-based false recognition in older adults: encoding and retrieval manipulations. *Psychol. Aging* 14:220. 10.1037//0882-7974.14.2.220 10403710

[B43] Litman L. Robinson J. Abberbock T. (2017). TurkPrime. com: a versatile crowdsourcing data acquisition platform for the behavioral sciences. *Behav. Res. Methods* 49 433–442. 10.3758/s13428-016-0727-z 27071389 PMC5405057

[B44] Long J. A. (2019). *Interactions: Comprehensive, User-Friendly Toolkit for Probing Interactions.* R package version 1.1. 0.

[B45] Lustig C. Hasher L. Zacks R. T. (2007). “Inhibitory deficit theory: recent developments in a “new view”,” in *Inhibition in Cognition*, eds Gorfein D. S. MacLeod C. M. (Massachusetts: American Psychological Association), 145–162. 10.1037/11587-008

[B46] Mata R. Schooler L. J. Rieskamp J. (2007). The aging decision maker: cognitive aging and the adaptive selection of decision strategies. *Psychol. Aging* 22:796. 10.1037/0882-7974.22.4.796 18179298

[B47] Moscovitch M. Craik F. I. M. (1976). Depth of processing, retrieval cues, and uniqueness of encoding as factors in recall. *J. Verbal Learn. Verbal Behav.* 15 447–458. 10.1016/s0022-5371(76)90040-2

[B48] Naveh-Benjamin M. (2000). Adult age differences in memory performance: tests of an associative deficit hypothesis. *J. Exp. Psychol. Learn. Mem. Cogn.* 26 1170–1187. 11009251 10.1037//0278-7393.26.5.1170

[B49] Naveh-Benjamin M. Hussain Z. Guez J. Bar-On M. (2003). Adult age differences in episodic memory: further support for an associative-deficit hypothesis. *J. Exp. Psychol. Learn. Mem. Cogn.* 29:826. 10.1037/0278-7393.29.5.826 14516216

[B50] Naveh-Benjamin M. Mayr U. (2018). Age-related differences in associative memory: empirical evidence and theoretical perspectives. *Psychol. Aging* 33 1–6. 10.1037/pag0000235 29494173

[B51] Old S. R. Naveh-Benjamin M. (2008). Differential effects of age on item and associative measures of memory: a meta-analysis. *Psychol. Aging* 23:104. 10.1037/0882-7974.23.1.104 18361660

[B52] Park D. C. Lautenschlager G. Hedden T. Davidson N. S. Smith A. D. Smith P. K. (2002). Models of visuospatial and verbal memory across the adult life span. *Psychol Aging* 17 299–320. 10.1037/0882-7974.17.2.299 12061414

[B53] Poppenk J. Köhler S. Moscovitch M. (2010). Revisiting the novelty effect: when familiarity, not novelty, enhances memory. *J. Exp. Psychol. Learn. Mem. Cogn.* 36:1321. 10.1037/a0019900 20804299

[B54] Preston A. R. Eichenbaum H. (2013). Interplay of hippocampus and prefrontal cortex in memory. *Curr. Biol.* 23 R764–R773. 24028960 10.1016/j.cub.2013.05.041 PMC3789138

[B55] Raz N. Lindenberger U. Rodrigue K. M. Kennedy K. M. Head D. Williamson A. (2005). Regional brain changes in aging healthy adults: general trends, individual differences and modifiers. *Cereb. cortex* 15 1676–1689. 10.1093/cercor/bhi044 15703252

[B56] Ren W. Li R. Zheng Z. Li J. (2015). Neural correlates of associative memory in the elderly: a resting-state functional MRI study. *Biomed Res. Int.* 2015 1–7. 10.1155/2015/129180 26180778 PMC4477061

[B57] Renoult L. Irish M. Moscovitch M. Rugg M. D. (2019). From knowing to remembering: the semantic–episodic distinction. *Trends Cogn. Sci.* 23 1041–1057. 10.1016/j.tics.2019.09.008 31672430

[B58] Rentz D. M. Amariglio R. E. Becker J. A. Frey M. Olson L. E. Frishe K. (2011). Face-name associative memory performance is related to amyloid burden in normal elderly. *Neuropsychologia* 49 2776–2783. 10.1016/j.neuropsychologia.2011.06.006 21689670 PMC3137730

[B59] Robin J. Olsen R. K. (2019). Scenes facilitate associative memory and integration. *Learn. Mem.* 26 252–261. 10.1101/lm.049486.119 31209120 PMC6581001

[B60] Roediger H. L. Meade M. L. Bergman E. T. (2001). Social contagion of memory. *Psychon. Bull. Rev.* 8 365–371. 11495127 10.3758/bf03196174

[B61] Salthouse T. A. (2003). *Interrelations of Aging, Knowledge, and Cognitive Performance,” in Understanding Human Development.* Berlin: Springer, 265–287.

[B62] Saverino C. Fatima Z. Sarraf S. Oder A. Strother S. C. Grady C. L. (2016). The associative memory deficit in aging is related to reduced selectivity of brain activity during encoding. *J. Cogn. Neurosci.* 28 1331–1344. 10.1162/jocn_a_00970 27082043 PMC4967371

[B63] Shing Y. L. Rodrigue K. M. Kennedy K. M. Fandakova Y. Bodammer N. Werkle-Bergner M. (2011). Hippocampal subfield volumes: age, vascular risk, and correlation with associative memory. *Front. Aging. Neurosci.* 3:2. 10.3389/fnagi.2011.00002 21331174 PMC3035014

[B64] Simonsmeier B. A. Flaig M. Deiglmayr A. Schalk L. Schneider M. (2022). Domain-specific prior knowledge and learning: a meta-analysis. *Educ. Psychol.* 57 31–54. 10.1080/00461520.2021.1939700

[B65] Spalding K. N. Jones S. H. Duff M. C. Tranel D. Warren D. E. (2015). Investigating the neural correlates of schemas: ventromedial prefrontal cortex is necessary for normal schematic influence on memory. *J. Neurosci.* 35 15746–15751. 10.1523/JNEUROSCI.2767-15.2015 26609165 PMC4659831

[B66] Staresina B. P. Gray J. C. Davachi L. (2009). Event congruency enhances episodic memory encoding through semantic elaboration and relational binding. *Cereb. Cortex* 19 1198–1207. 10.1093/cercor/bhn165 18820289 PMC2665161

[B67] Stawarczyk D. Wahlheim C. N. Etzel J. A. Snyder A. Z. Zacks J. M. (2020). Aging and the encoding of changes in events: the role of neural activity pattern reinstatement. *Proc. Natl. Acad. Sci.U.S.A.* 117 29346–29353. 10.1073/pnas.1918063117 33229530 PMC7703536

[B68] Tromp D. Dufour A. Lithfous S. Pebayle T. Després O. (2015). Episodic memory in normal aging and Alzheimer disease: insights from imaging and behavioral studies. *Ageing Res. Rev.* 24 232–262. 10.1016/j.arr.2015.08.006 26318058

[B69] Tuckey M. R. Brewer N. (2003). The influence of schemas, stimulus ambiguity, and interview schedule on eyewitness memory over time. *J. Exp. Psychol. Appl.* 9:101. 10.1037/1076-898x.9.2.101 12877270

[B70] Tulver K. Aru J. Rutiku R. Bachmann T. (2019). Individual differences in the effects of priors on perception: a multi-paradigm approach. *Cognition* 187 167–177. 10.1016/j.cognition.2019.03.008 30877848

[B71] Umanath S. Marsh E. J. (2014). Understanding how prior knowledge influences memory in older adults. *Perspect. Psychol. Sci.* 9 408–426. 10.1177/1745691614535933 26173273

[B72] van Kesteren M. T. R. Rignanese P. Gianferrara P. G. Krabbendam L. Meeter M. (2020). Congruency and reactivation aid memory integration through reinstatement of prior knowledge. *Sci. Rep.* 10 1–13. 10.1038/s41598-020-61737-1 32179822 PMC7075880

[B73] Van Kesteren M. T. R. Ruiter D. J. Fernández G. Henson R. N. (2012). How schema and novelty augment memory formation. *Trends Neurosci.* 35 211–219. 10.1016/j.tins.2012.02.001 22398180

[B74] Verhaeghen P. Salthouse T. A. (1997). Meta-analyses of age-cognition relations in adulthood: estimates of linear and nonlinear age effects and structural models. *Psychol Bull* 122 231–249. 10.1037/0033-2909.122.3.231 9354147

[B75] Webb C. E. Dennis N. A. (2020). Memory for the usual: the influence of schemas on memory for non-schematic information in younger and older adults. *Cogn. Neuropsychol.* 37 58–74. 10.1080/02643294.2019.1674798 31583953 PMC8919503

[B76] Wynn J. S. Ryan J. D. Moscovitch M. (2020). Effects of prior knowledge on active vision and memory in younger and older adults. *J. Exp. Psychol. Gen.* 149:518. 10.1037/xge0000657 31343184

[B77] Yee E. Chrysikou E. G. Thompson-Schill S. L. (2014). “Semantic memory,” in *The Oxford Handbook of Cognitive Neuroscience*, Vol. 1 eds Ochsner K. N. Kosslyn S. M. (Oxford: Oxford University Press), 353–374.

