# Supplementary figures and images for: A Double-Edged Sword: The Role of Prior Knowledge in Memory Aging
Source: Front Aging Neurosci. 2022 May 10;14:874767. doi: 10.3389/fnagi.2022.874767 (PMC9127270; doi:10.3389/fnagi.2022.874767)

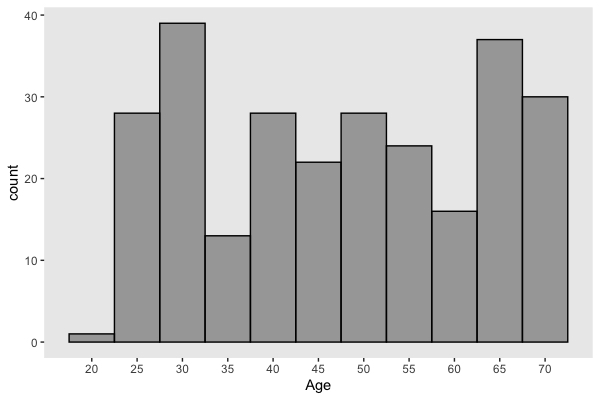

Supplement: Supplementary Figure 1 — Age distribution of the sample. [file Image_1.JPEG]

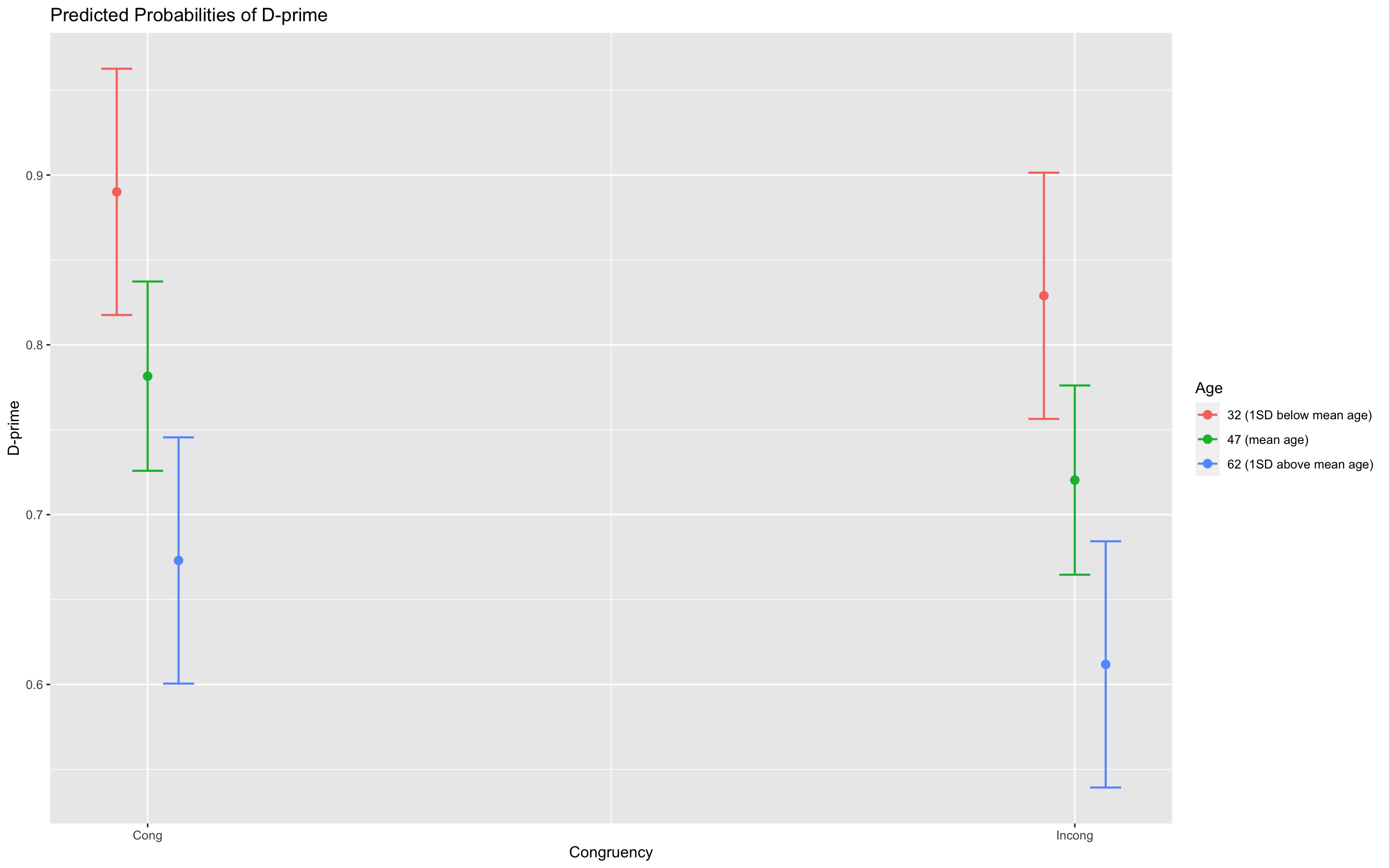

Supplement: Supplementary Figure 2 — Higher congruency marginally related to better memory discrimination. [file Image_2.JPEG]
